# The Friction–Lubrication Effect and Compaction Characteristics of an SMA Asphalt Mixture under Variable Temperature Conditions

**DOI:** 10.3390/ma17071694

**Published:** 2024-04-07

**Authors:** Xia Wu, Xiong Tang, Li Liu, Zhaoyi He, Sheng He

**Affiliations:** 1 School of Civil Engineering, Chongqing Jiaotong University, Chongqing 400074, China; xswuxia1@jxyy.edu.cn (X.W.);; 2 School of Architecture and Engineering, Jiangxi College of Applied Technology, Ganzhou 341000, China; 3 Sichuan Transportation Construction Group Co., Ltd., Chengdu 610047, China

**Keywords:** stone mastic asphalt, compaction degree, compaction energy index, friction–lubrication effect

## Abstract

The aim of this article is to explore the dynamic compaction characteristics of stone mastic asphalt (SMA) and the friction–lubrication effect of internal particles during the superpave gyratory compaction (SGC) process. Firstly, a calculated method for the compaction degree of an asphalt mixture in the gyratory compaction process was defined based on the multiphase granular volume method. Secondly, the gyratory compaction curves of asphalt mixtures were taken based on this calculation method of compaction degree. The dynamic change law of each compaction index (compaction, percentage of air voids, compaction energy index, etc.) during the compaction process was analysed. Finally, the effects of different initial compaction temperatures and different asphalt content on the friction–lubrication effect and compaction characteristics of asphalt mixtures were studied. Research shows that it is reasonable to define the compaction degree by the ratio of the apparent density of the asphalt mixture to the maximum theoretical density of the asphalt mixture during gyratory compaction. The dynamic prediction equations of the compaction degree K and the compaction energy index CEI with the amount of compaction were established, and could effectively predict the compaction degree, percentage of air voids and compaction energy index CEI. The compaction process of the asphalt mixture needed to go through three phases, including periods of rapid growth, slow growth, and stabilisation, and the compaction degree increased by about 10%, 5%, and 1%, in that order, finally tending towards a stable value. The effect of the initial compaction temperature on the forming compaction degree of the asphalt mixture is significant; therefore, it should be controlled strictly in the compaction construction of asphalt mixtures. When the initial compaction temperature of SMA-13 is about 170 °C, the compaction effect is optimal, and the effect of the increase in the amount of compaction at a later stage on the increase in the compaction degree of the asphalt mixture is very low. With the optimal asphalt content, the friction–lubrication effect between the asphalt and aggregate particles is optimal, because it can effectively form an asphalt film, reducing the frictional resistance of the particles moving each other during the compaction process, and the voids will be embedded and filled with each other, finally producing the best compaction result.

## 1. Introduction

Compaction is a key process to ensure the construction quality of asphalt pavements [1]. The degree of compaction is an important evaluation index for compaction quality and affects the life and performance of asphalt pavements [2]. Stone mastic asphalt is widely used as a wearing course in pavement projects due to its good performance [3,4]. Asphalt mixtures are multi-phase particle aggregates, and the physical properties of asphalt mixtures are defined by the multi-phase volumetric method [5,6,7]. The compaction degree of asphalt mixtures is mainly defined and calculated in laboratory experiments based on its physical properties (such as density, apparent density, and specific gravity) [6,8]. The particles of asphalt mixtures are mainly vertically transmitted under the action of the roller during on-site compaction, and the SGC test can simulate the compaction process more effectively [9,10]. The compaction characteristics of asphalt mixtures mainly include compaction degree, percentage of air voids, and compaction energy index (CEI) [11,12]. The interaction of internal particles of asphalt mixtures is mainly through contact pressure during the compaction process; the increase rate of the compaction degree and compaction energy is large at the initial stage, and becomes smaller gradually at the later stage [13]. There is a critical point in the compaction process of asphalt mixtures at which the skeleton structure becomes stable and the compaction degree tends to stabilise beyond the critical point [14]. The critical point can be determined by the sample height and density changes in the SGC test, which for structural stability for SMA is relatively stable [15,16]. The compaction energy index (CEI) correlates well with the compaction energy and structural stability [17,18]. The effects of gradation, initial compaction temperature, and asphalt content on compaction characteristics were significant [19,20]. During the compaction process, as the initial compaction temperature decreases, the action of asphalt changes from a lubricating force to a cementing force, the movement speed between particles slows down, and the friction force increases [21]. The percentage of air voids in the asphalt mixture decreases with the increase in the amount of coarse aggregate grading, and the asphalt mixture which uses intermittent grading is favourable to increase the compaction of the mixture [22]. Higher asphalt content or higher compaction temperatures lead to a reduction in the contact strength of the asphalt mixture, which improves its work ability [23]. During compaction, the friction–lubrication effects between aggregate particles have a significant influence on the compaction characteristics and compaction effect; for example, sufficiently lubricated dense-graded asphalt mixtures are easily compacted [24]. In addition, some scholars have defined some new indicators to describe the compaction properties [25]; for example, based on the variation in particle stress and the height of the specimen, the rate change of compaction in logarithmic coordinates has been proposed to characterise the compaction properties of asphalt mixtures [26,27]. The compaction increase rate (CIR) indicator was defined to describe the quality of pre-compaction based on discrete element method (DEM) simulation and field tests [28].

Previous studies have been positive in providing a deeper understanding of the compaction process for asphalt mixtures. However, the above studies mainly focus on static and qualitative analyses of compaction characteristics and compaction indexes during the gyratory compaction of asphalt mixtures, with fewer dynamic changes and quantitative studies on compaction curves, compaction indexes (degree of compaction, percentage of air voids, and compaction energy index, etc.), and fewer studies on the effects of asphalt dosage and initial compaction temperature on the friction–lubrication effect of asphalt mixtures.

Therefore, establishing a good correlation between the compaction curve, compaction index, and friction–lubrication effect, and undertaking a dynamic and quantitative analysis of the compaction characteristics and related indicators, will help to provide an in-depth understanding of the compaction mechanism of asphalt mixtures, a stronger basis for effective on-site construction control in compaction processes, and compaction quality assurance.

## 2. Materials and Methods

### 2.1. Materials

Stone mastic asphalt (SMA) is mainly composed of asphalt, aggregates (coarse and fine), mineral fillers, and fibres. The coarse and fine aggregates used in the asphalt mixture in the experiments were basalt from Sichuan, China. The coarse and fine aggregates, mineral filler and fibres’ physical specifications are shown in Table 1, Table 2, Table 3 and Table 4 [29]. The asphalt of SMA in the experiments is SBS-modified asphalt, and its properties are shown in Table 5 [30].

The relationship between density and specific gravity is as follows in Table 1, Table 2, Table 3, Table 4 and Table 5: The apparent specific gravity is the ratio of apparent density to the density of water at the same temperature, and is thus dimensionless. Bulk specific gravity is the same. The specific gravity of the mineral filler, wood fibre, and SBS-modified bitumen was measured using experiments, and the densities were back-calculated according to the density of water and the specific gravity.

In order to study the friction response and compaction characteristics between coarse and fine aggregate during the compaction process of SMA, the aggregate gradation of SMA-13 is as follows in Table 6.

### 2.2. Definition of the Compaction Degree of SMA in Gyratory Compaction

Compaction degree (K) is an evaluation indicator to reflect the asphalt mixture compaction quality. In the indoor experiments, the compaction degree (K) of the asphalt mixture was the ratio of the bulk specific gravity to the maximum theoretical specific gravity for the specimen (dimensionless).

**Figure 1:**
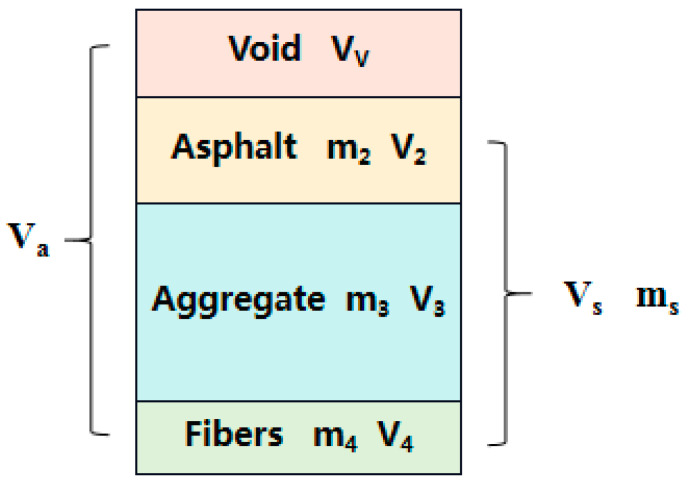
SMA asphalt mixture multiphase system.

This indicator only reflects the compaction degree of the moulding asphalt mixture, and fails to reflect the change in compaction status during the compaction process. In order to analyse the compaction characteristics of the gyratory compaction process, stone mastic asphalt (SMA) is viewed as a collection of particles (asphalt, aggregate, and fibres), and the multi-phase volumetric method can be used to define the degree of compaction of SMA in the gyratory compaction, as shown in Figure 1.

Here, $m_{2}$ is the mass of the asphalt (g), $m_{3}$ is the mass of the aggregate (g), $m_{4}$ is the mass of the fibres (g), $m_{s}$ is the total mass of the asphalt mixture (g); $V_{V}$ is the void volume (cm^3^), $V_{2}$ is the volume of the asphalt (cm^3^), $V_{3}$ is the volume of the aggregate (cm^3^), $V_{4}$ is the volume of the fibre stabilizer (cm^3^); $V_{a}$ is the total volume of the asphalt mixture (cm^3^), and $V_{s}$ is the real volume of the asphalt mixture (cm^3^). According to the mechanical theory of granular materials, the following relationships can be defined as follows:

(1) $$V_{a}=V_{V}+V_{s}$$

(2) $$\rho_{s}=\frac{m_{s}}{V_{s}}$$

(3) $$\rho_{a}=\frac{m_{s}}{V_{a}}$$

In Formulas (1)–(3), $\rho_{s}$ is the maximum theoretical density of the asphalt mixture (g/cm^3^) and $\rho_{a}$ is the apparent density of the asphalt mixture (g/cm^3^).

In order to characterise the compaction properties of gyratory compaction, the compaction degree (K) is introduced based on Figure 1, as follows:

(4) $$K=\frac{\rho_{a}}{\rho_{s}}\times 100\%$$

where $\rho_{s}$ can be calculated by Equations (5) and (6):

(5) $$\rho_{s}=\gamma_{s}\times \rho_{w}$$

(6) $$\gamma_{s}=\frac{100+\omega +P_{x}}{\frac{\omega }{\gamma_{a}}+\frac{P_{x}}{\gamma_{x}}+\frac{100}{\gamma_{se}}}$$

where $\gamma_{s}$ is the maximum theoretical specific gravity of the asphalt mixture (dimensionless); $\rho_{w}$ is the density of water at room temperature, 25 °C (g/cm^3^), which is taken as 0.9971 g/cm^3^; $P_{x}$ is the fibre content (%); ω is the asphalt aggregate ratio (%); $\gamma_{a}$ is the specific gravity of asphalt (dimensionless), $\gamma_{x}$ is the fibre’s specific gravity (dimensionless); and $\gamma_{se}$ is the aggregate’s specific gravity (dimensionless), which can be calculated by measuring the specific gravity of each grade of aggregate.

A variable *i* is introduced to represent the *i*-th gyratory compaction. According to Equation (4), the compaction degree of the *i*-th gyratory compaction ($K_{i}$) can be written as follows:

(7) $$K_{i}=\frac{\rho_{i}}{\rho_{s}}\times 100\%$$

(8) $$\rho_{i}=\frac{m_{s}}{V_{i}}=\frac{m_{s}}{\pi R^{2}H_{i}}$$

where $\rho_{i}$ is the apparent density of the *i*-th gyratory compaction (g/cm^3^), $\rho_{s}$ is the maximum theoretical density of the asphalt mixture (g/cm^3^), $m_{s}$ is the mass of the gyratory compaction specimen in the air (g), *R* is the radius of the gyratory compaction test mould (cm), and $H_{i}$ is the height of the *i*-th gyratory compaction specimen (cm). Based on Equations (5)–(8), the $K_{i}$ of SMA can be calculated according to changes in specimen height, thus providing a compaction curve that varies with the amount of compaction.

### 2.3. SGC Test

The superpave gyratory compaction (SGC) test is an indoor test method based on repeated kneading under vertical pressure to simulate the on-site compaction and rolling effect of asphalt mixtures. The gyratory compactor mainly consists of a reaction frame, a loading device, a rotating base, a control system, an internal rotation angle measuring device, a test mould, a hammer head and a base, a force measuring device, and a pressure sensing device. It can also be equipped with a shearing device. Stress testing systems measure shear stress during compaction.

**Figure 2:**
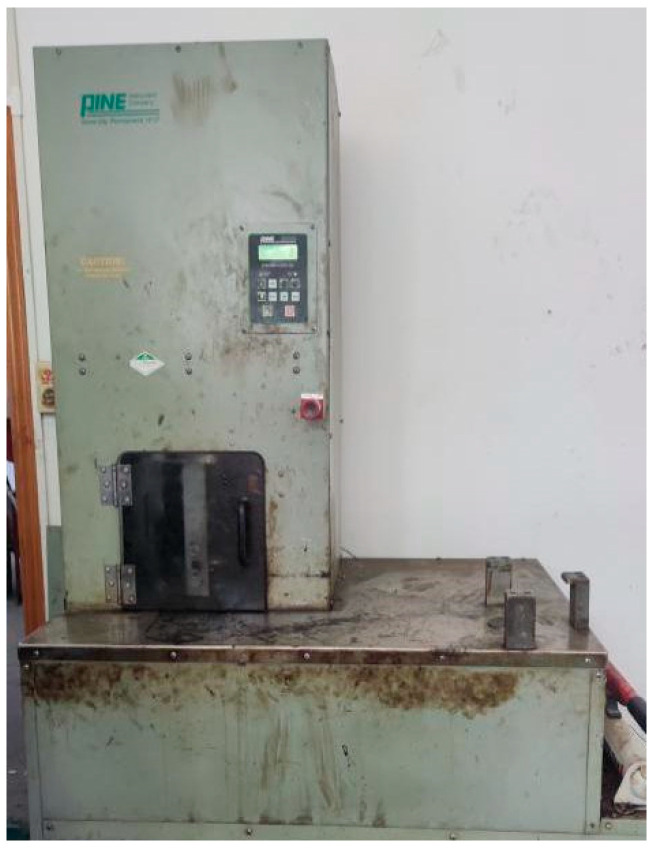
Superpave gyratory compactor.

**Figure 3:**
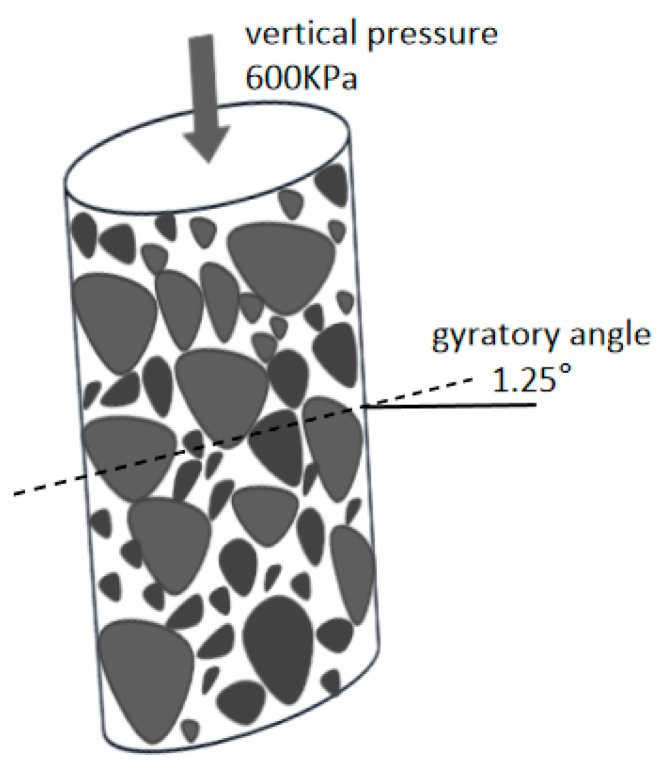
Working principle of the SGC.

The basic principle of the SGC compactor: the specimen is slowly compacted under the vertical pressure of a loading head of 600 KPa. Its movement axis is like a cone, with its apex coinciding with the top of the specimen. The rotating base positions the test mould at 1.25°. The gyratory compaction angle rotates at a constant speed of 30 r/min. The loose asphalt mixture is simultaneously affected by vertical pressure and horizontal shear force in the test mould, causing the particles to move directionally to form a dense skeleton structure. The gyratory compactor and its working principle are shown in Figure 2 and Figure 3.

## 3. Results and Discussion

### 3.1. Establishing a Compaction Prediction Equation

**Figure 4:**
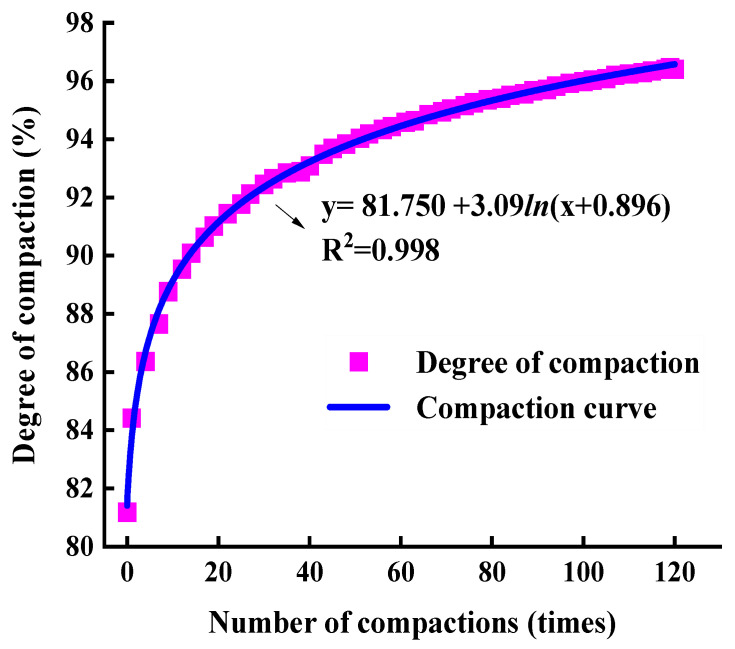
Compaction curve of SMA-13.

In the SGC test, the bottom and sides of the mould have lateral and vertical constraints on the asphalt mixture, but there is no constraint on the top. Therefore, according to Equation (8), the degree of compaction during the gyratory compaction process can be taken based on the change in the specimen height. In order to analyse the relationship between the amount of gyratory compaction of the asphalt mixtures and the degree of compaction, a logarithmic function is used to fit the degree of compaction. The compaction curve of the SMA-13 asphalt mixture can be taken as shown in Figure 4.

Figure 4 shows that the fitting equation of the compaction curve of SMA-13 is as follows:

y=81.75+3.09ln(x+0.896)

where y is the degree of compaction (%) and x is the amount of gyratory compaction (times). $R^{2}$ is the goodness of fit. The closer its value is to 1, the closer the fitting curve is to the true distribution of the data. When $R^{2}$ is 0.998, this indicates that the fitting equation can describe the degree of compaction well. The compaction curve in Figure 4 shows a good logarithmic function relationship between the compaction degree and the amount of compaction, which can accurately describe the changing trend of the compaction degree during the SGC process of asphalt mixtures.

Therefore, the estimated equation of the gyratory compaction curve can be summarized as follows:

(9) $$K_{i}=A+Bln(i+C),\;i=1,\;2,\;3,\;\ldots \ldots I_{des}$$

where $K_{i}$ is the compaction degree of the i-th gyratory compaction of the asphalt mixture, A, B, and C are the fitting coefficients, i is the i-th amount of gyratory compaction, and $I_{des}$ is the corresponding amount of gyratory compaction when the compaction degree is qualified and stable. The average of the fitted data from multiple tests is taken as the value of the fitting coefficients. Based on predictive Equation (9), the compaction degree that varies with the amount of compaction can be estimated, which will greatly reduce the workload of the test parameter calibration and also provide a certain reference point for the on-site prediction of the compaction degree.

### 3.2. Establishing a Compaction Energy Prediction Equation

The compaction energy index (CEI) refers to the work undertaken by the compaction load on the asphalt mixture during the gyratory compaction process. In this process, the asphalt mixture is gradually compacted and the void ratio is continuously reduced. CEI is related to the compaction energy. CEI can be represented by the integral area (S) of the curve between any two points ($X_{1},X_{2}$) on the compaction curve. The calculation of CEI is as follows:

(10) $$CEI=\int_{x_{1}}^{x_{2}}f(x)dx$$

**Figure 5:**
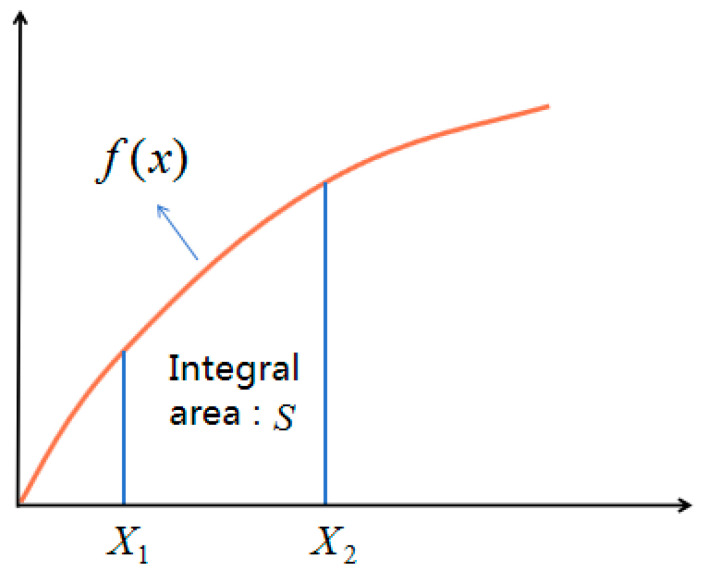
Calculation diagram of CEI.

Figure 5 is the calculation diagram of CEI, as follows.

Here, y is the value of CEI (dimensionless), x is the amount of gyratory compaction (times), and f(x) is the curve of compaction. Based on Equations (9) and (10), CEI can be expressed as the following equation:

(11) $$CEI=\int_{I_{1}}^{I_{2}}A+B\ln(i+C)d_{i}$$

where *i* is a variable for the amount of compaction. Figure 5 shows that the lower the CEI, the lower the energy required for the target compaction degree, the better the compact ability, and the higher the compaction efficiency.

**Figure 6:**
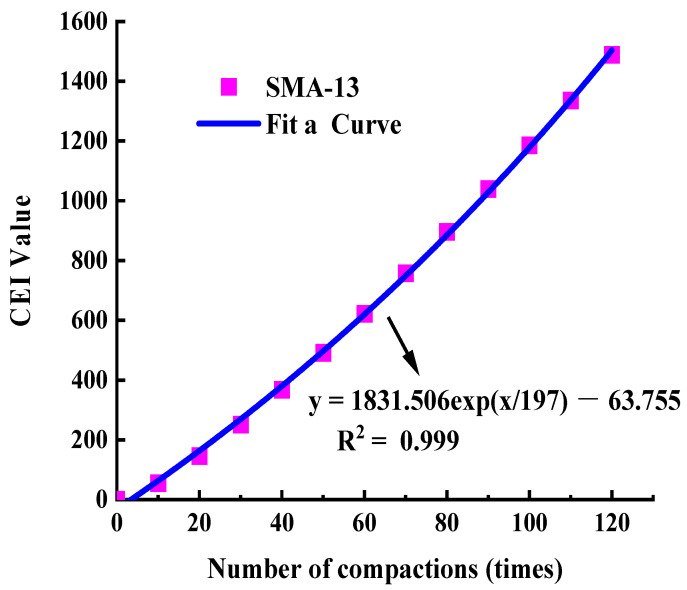
CEI curve of SMA-13.

Based on the compaction curve in Figure 4 and Equation (11), the work undertaken can be calculated for the asphalt mixture from the 0-th to the *i*-th compaction, that is, the CEI value of the first *i* times. In order to explore the changing rules of the CEI value during the gyratory compaction process, the CEI values from the 0th to the 10th, 20th, 30th, ‖, 120th time were obtained, which covered the total work undertaken by the asphalt mixture at this number of compaction times. The results are shown in Figure 6.

In Figure 6, the curve was fitted based on the data and the curve function expressions are as follows:

$$y=1831.506e^{\frac{x}{197}}-1863.755$$

where y is the compaction energy index (CEI) (dimensionless); x is the amount of compaction (times); and R² is the goodness of fit. When $R^{2}$ is 0.999, this indicates that the fitting equation can describe the relationship between the compaction energy index CEI and the compaction times well. There is a good exponential function relationship between the compaction energy and the amount of compaction, which can accurately describe the changing trend of the CEI during the compaction process of the asphalt mixture.

Therefore, the estimated equation of the CEI curve can be summarized as follows:

(12) $$CEI_{i}=Ae^{Bi}+C,\;i=1,\;2,\;3,\;\ldots \ldots I_{des}$$

where $CEI_{i}$ is the total work undertaken by the first *i* gyratory compaction times of the asphalt mixture; A, B, and C are the fitting coefficients; i is the amount of gyratory compaction; and $I_{des}$ is the corresponding amount of compaction when the compaction degree is qualified and stable. The average of the fitted data from multiple tests is taken as the value of the fitting coefficients.

Based on predictive Equation (12), the CEI value with different amounts of compaction can be estimated, which will provide a certain reference point for the on-site prediction of compaction energy.

### 3.3. Effect of Initial Compaction Temperature on Inter-Particle Friction

**Figure 7:**
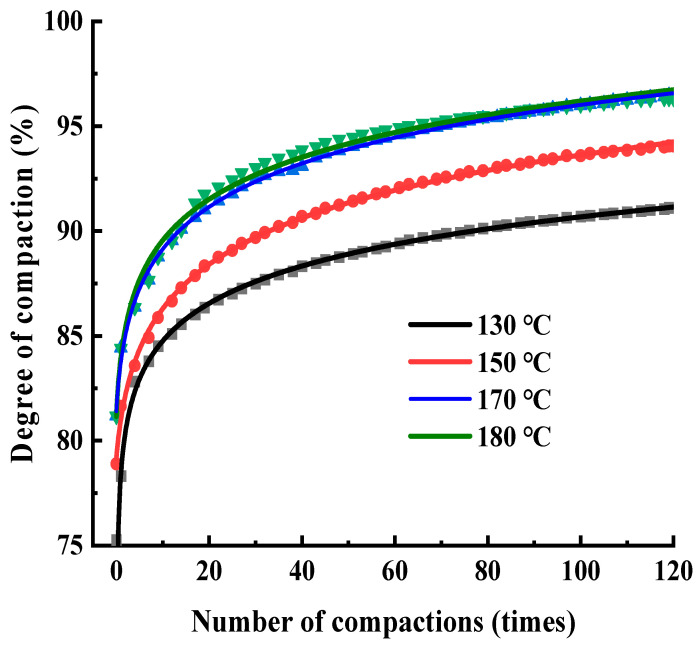
Gyratory compaction curves at different initial compaction temperatures.

The gyratory compaction curve can better reflect the internal structural characteristics of the asphalt mixture during compaction. The compaction tests of variable temperature involve changing the initial compaction temperature ($T_{0}$) to explore the changing rules of the compaction degree, structural percentage of air voids, and the friction response between the internal particles. SMA-13 is selected, and the gradation is as shown in Table 6, above. The asphalt is 90^#^ SBS modified bitumen. The optimal content is 5.8%. In order to simulate the aging of the construction process, the asphalt is placed in a high-temperature oven for more than 2 h. The initial compaction temperature $T_{0}$ of the test is set to 130 °C, 150 °C, 170 °C, and 180 °C, and the number of gyratory compactions is 120 times. Three specimens are taken from each group, and the compaction degree is calculated using Equation (8). The relative error of the compaction degree is less than 0.3%, and the average of the three groups of compaction degrees is taken. Nonlinear fitting was performed based on the SGC logarithmic function fitting Equation (9), and the fitting degree was >99.8%. The compaction curves under different initial compaction temperatures are shown in Figure 7.

Figure 7 shows the compaction curves of SMA-13 at different initial compaction temperatures, and the trend of different compaction curves is more or less the same. Changes in the degree of compaction directly reflect changes in the porosity of the internal structure, and the degree of compaction (K) has the following relationship with the void ratio (VV):

(13) $$\Delta K=K_{1}-K_{2},\;\Delta VV=VV_{1}-VV_{2}$$

(14) $$K_{1}+VV_{1}=K_{2}+VV_{2}=1(100\%)$$

(15) Thus ΔK=−ΔVV

where VV is the percentage of air voids in the asphalt mixture (%); there is a negative correlation between K and VV.

**Figure 8:**
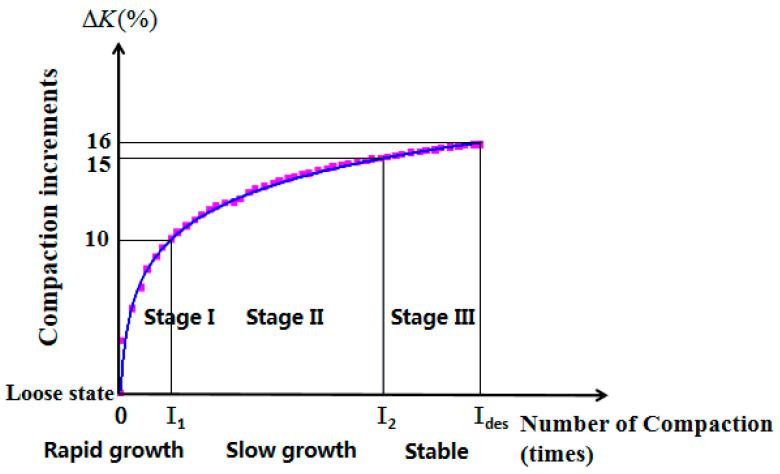
Schematic diagram of the compaction growth stages.

Analysing the compaction curve in Figure 7, it can be seen that the compaction degree growth is rapid in the early stage, during gyratory compaction, slows down in the middle stage, and tends to be stable in the later stage. In order to better analyse the compaction process, this paper proposes to divide the gyratory compaction process into three compaction stages: Stage I is the rapid growth period, where the compaction degree grows by about 10%, which corresponds to I_1_ times of compaction, and the interval of the number of times of compaction is (0, 20); Stage II is the slow growth period, where the compaction degree grows by about 5%, which corresponds to I_2_ times of compaction, and the interval of the number times of compaction is (20, 90); Stage III is a stable period, where the compaction degree grows by about 1%, and the interval of the number of times of compaction is (90, I_des_), finally trending to a stable value, with a compaction degree growth of about 15–16% during compaction. The compaction degree increments of the gyratory compaction curve are shown in Figure 8.

**Figure 9:**
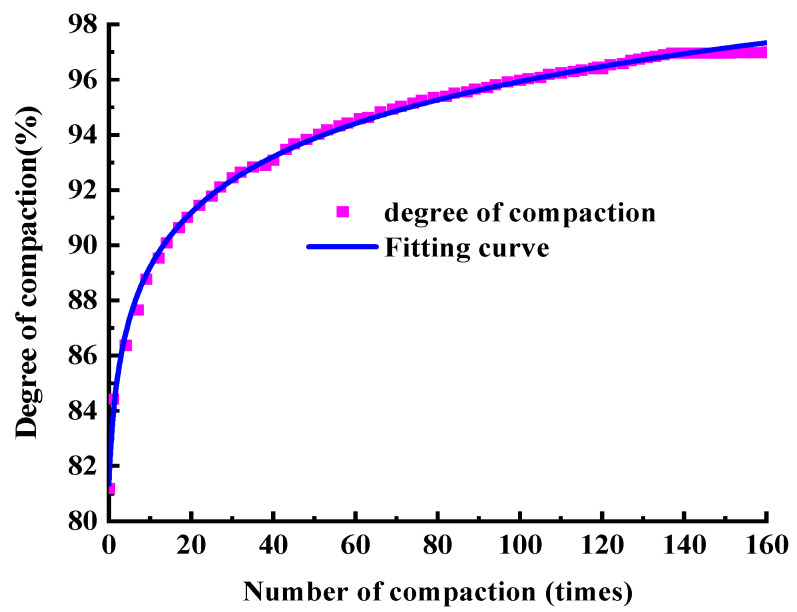
Compaction curve of SMA-13.

In order to verify the accuracy of the compaction stage prediction for the asphalt mixture, the amount of compaction is set at 160 times, the initial compaction temperature is 170 °C in the SGC test, and the compaction curve of SMA-13 is shown in Figure 9. The figure shows that the stage of the compaction degree growth is set reasonably, and the compaction degree tends to converge horizontally after 120 compaction times, which verifies the reasonableness of the compaction stage grading.

**Figure 10:**
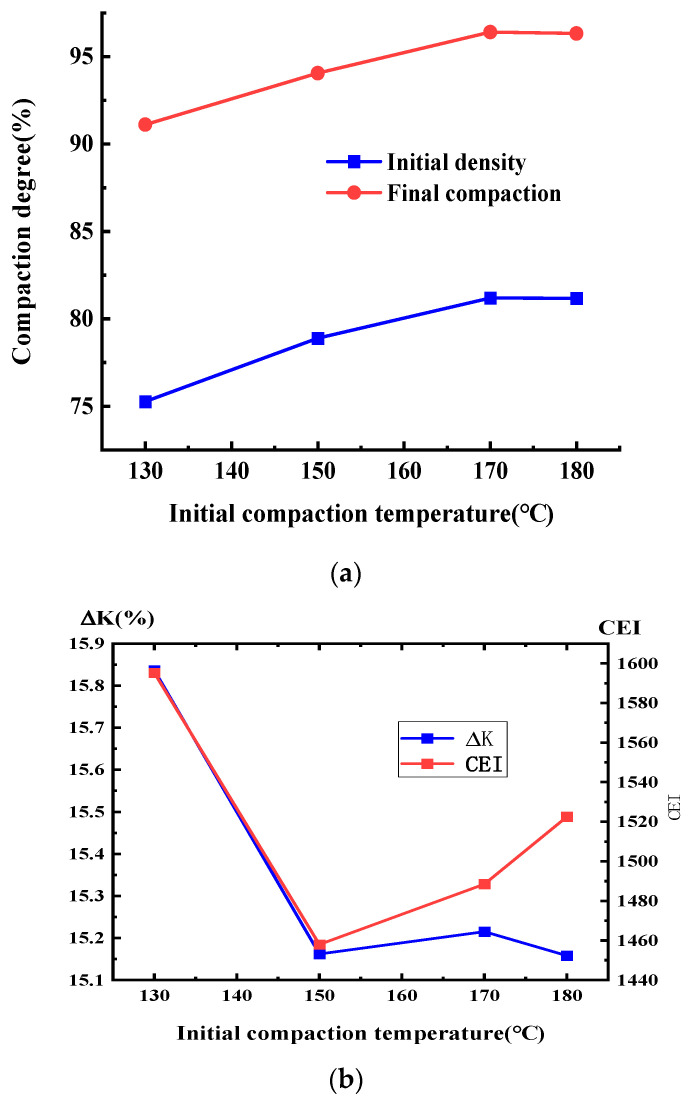
Variation in compaction index for SMA-13 under variable temperatures. (**a**) Initial and final compaction degree. (**b**) Compaction degree increments and CEI.

The initial density, final compaction degree, compaction degree increment, and CEI of SMA were measured at different compaction temperatures, and the results are shown in Figure 10.

Figure 10 shows the total increment of the compaction degree under different initial compaction temperatures for a certain compaction load; the total amount of compaction and gradation did not change much, due to the height change in the specimen being basically the same in the gyratory compaction of the asphalt mixtures, which indicates that the total relative displacement between the particles in the compaction process of asphalt mixtures under different initial temperatures is approximately the same.

**Figure 11:**
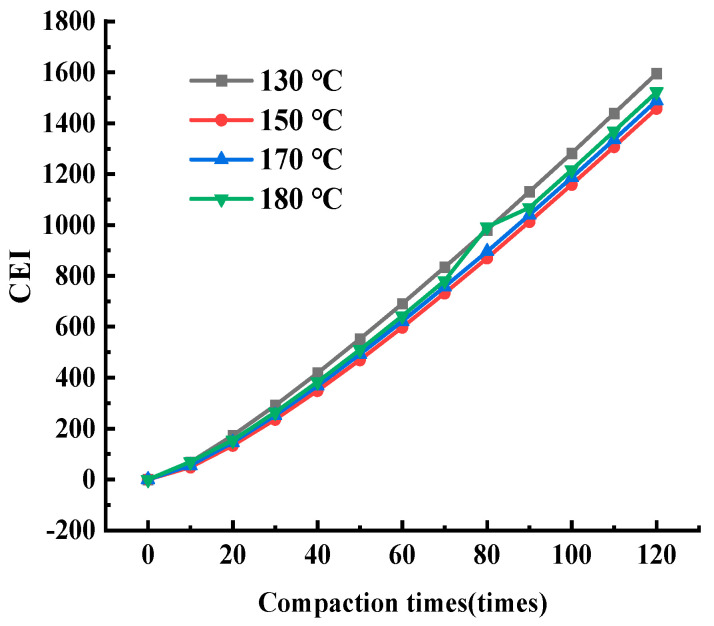
Variation in CEI for SMA-13 under variable temperatures.

CEI curves based on Formula (12) are shown under different compaction temperatures in Figure 11. The final CEI and compaction degree increments are shown in Figure 10b, and the CEI of the asphalt mixture at different initial temperatures did not change significantly, which indicates the work undertaken to overcome the frictional resistance during compaction. Figure 10a shows that the initial density and the final compaction degree display greater difference when the total amount of compaction is constant.

The initial compaction temperature has a significant effect on the percentage of air voids and the compactness of loose asphalt mixtures. Because the compaction temperature has a significant effect on the lubrication effect of the asphalt film wrapped over the aggregate, when the initial compaction temperature is low, the viscosity of the asphalt decreases, the mobility is slow, the coarse and fine aggregates wrapped around the asphalt cannot slide effectively, and the fine aggregates cannot fill the larger gaps between the coarse aggregates, which leads to a larger percentage of air voids in the loose asphalt mixture and a smaller increase in the late compaction degree of the gyratory compaction, or even a failure to meet the compaction requirements.

Figure 8, Figure 9 and Figure 10 show that it is difficult to increase the compaction degree even when the amount of compaction is increased at a later stage, which further indicates that the effect on the improvement of the compaction quality is low when increasing the compaction work or the amount of compaction in later compaction stages.

However, for the compaction temperature it is not a case of the higher the better; when the temperature reaches 180 °C, the compaction degree of SMA asphalt mixtures is reduced, because when the asphalt mixture temperature is too high, the asphalt ages easily, the properties of the aging asphalt change, and the lubrication effect of the asphalt mixtures is reduced. Therefore, the initial compaction temperature during the final compaction degree of asphalt mixtures has a significant impact on the field compaction process, and strict attention should be paid to the control of the initial compaction temperature, with the rolling followed closely, which will allow poor compaction quality to be avoided. The initial compaction temperature is recommended as a control indicator of the field compaction process to ensure the compaction quality of the asphalt mixtures.

### 3.4. Influence of Asphalt Content on the Particle Lubrication–Friction Effect

**Figure 12:**
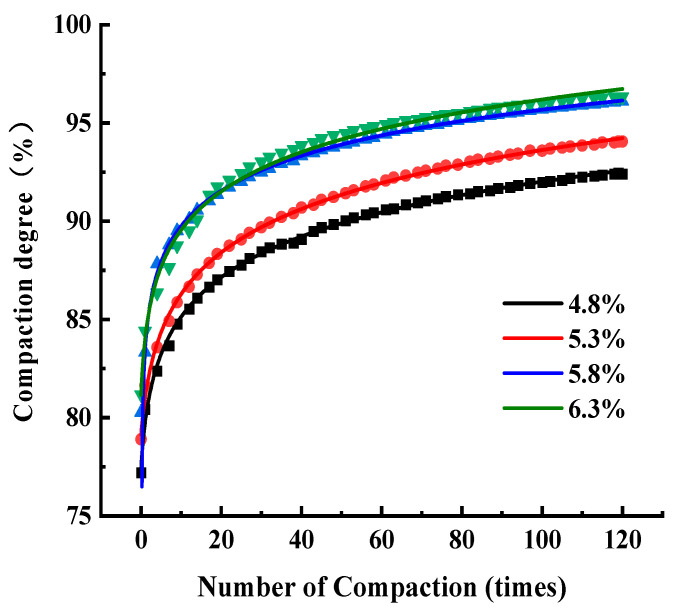
Compaction curves of SMA-13 with differing asphalt content.

In order to study the effect of asphalt content on the compaction characteristics of SMA-13, SBS 90^#^ modified bitumen was used in the test. The initial compaction temperature was set to the optimum temperature, 170 °C, the SMA-13 gradation was as shown in Table 6, the asphalt content was designed for 4.8%, 5.3%, 5.8%, and 6.3%, and the number of gyratory compaction was 120 times; the compaction curves are shown in Figure 12.

**Figure 13:**
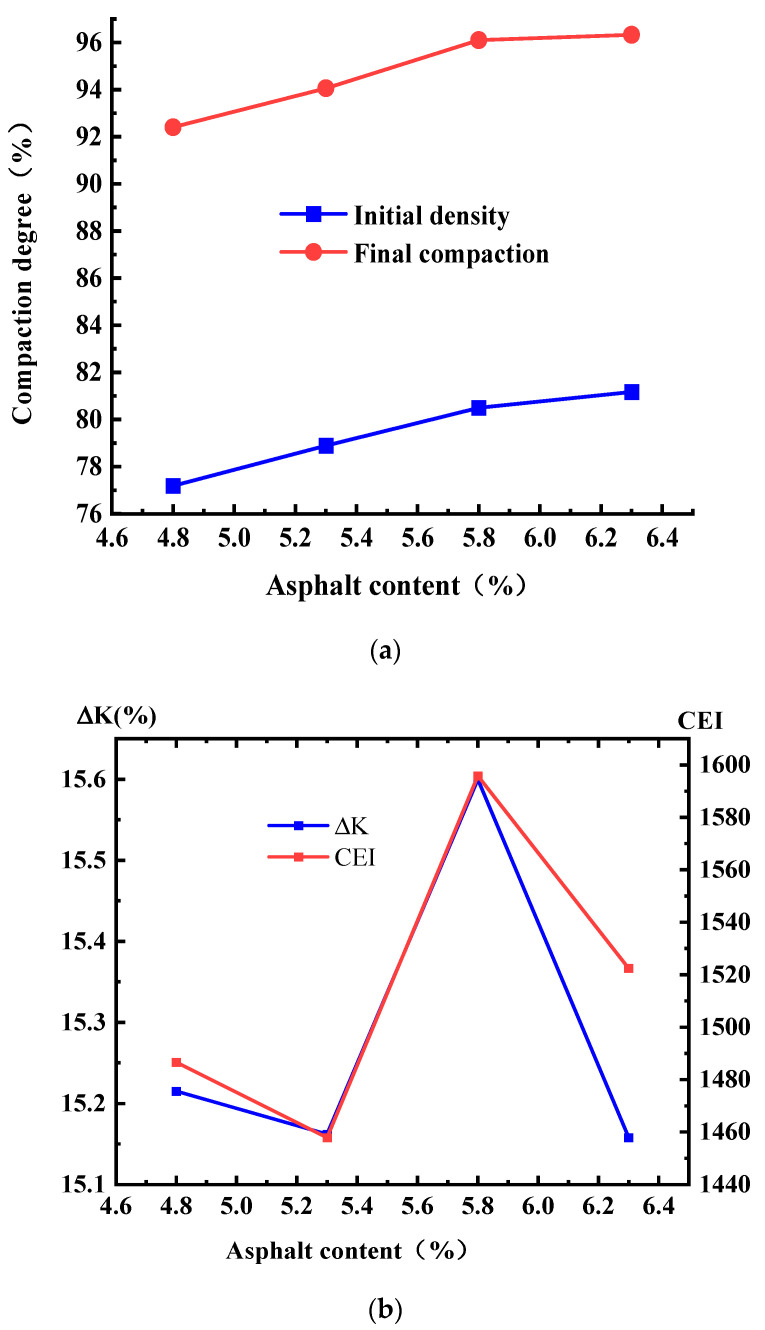
Variation in compaction index of SMA-13. (**a**) Initial density and final compaction degree. (**b**) Compaction increments and CEI values.

**Figure 14:**
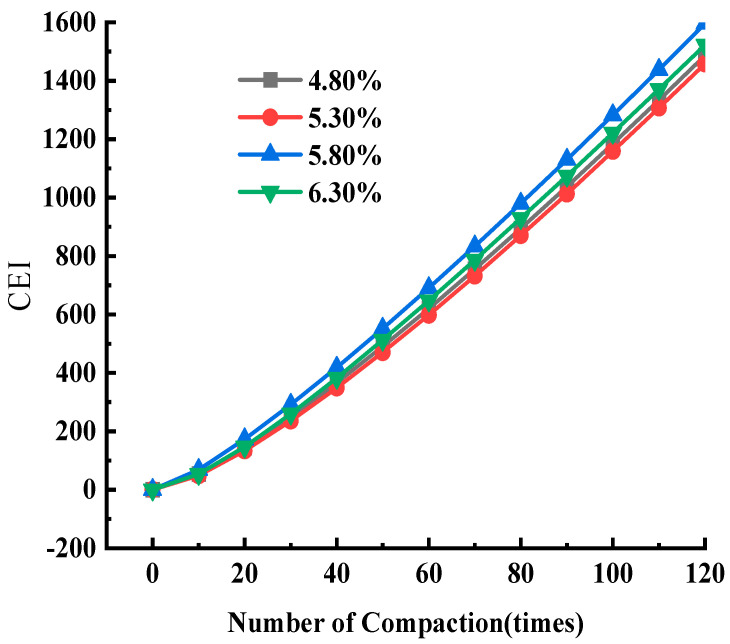
CEI curve for SMA-13 with differing asphalt content.

The initial density, final compaction degree, compaction degree increment, and CEI of SMA were measured at different asphalt contents, and the results are shown in Figure 13. The CEI curves of SMA at different asphalt contents are shown in Figure 14.

Figure 12 shows that when the asphalt type and gradation are certain, the compaction degree will increase with the increase in asphalt content. Due to the higher asphalt content, asphalt can enhance the lubrication effect between particles and the formation of a complete asphalt film, and the lubricating force between particles of the asphalt mixture will increase, making it easy to slide between particles. High asphalt content affected the friction between particles, reducing the frictional resistance of the particles to be filled. Particles were embedded with each other, which made compaction easy, and the particles were more fully in contact with each other, allowing a higher degree of compaction to be obtained. However, asphalt content is not a case of the higher the better, and when the asphalt content exceeds the optimal asphalt content, the asphalt forms a thicker oil film, and under the action of load and high temperature, the excess free asphalt will flow along the voids and seep out to the surface, resulting in oiling and rutting of the pavement, which affects the service life of the asphalt pavement. 

Figure 13 shows that both initial and final compaction increase with increasing asphalt content, and the maximum value of incremental compaction was achieved when the asphalt content was 5.8%. Figure 14 and Figure 13b show that the CEI values of asphalt mixtures at different asphalt contents do not vary significantly, and the work done to overcome the frictional resistance varies comparably during compaction, and the CEI is maximum at an asphalt content of 5.8%, which indicates that the most work is done by gyratory compaction of the asphalt mixture at this asphalt content, the maximum relative displacement is generated between the particles, and the friction-lubrication effect is optimal.

Therefore, this graded asphalt mixture has the best compaction effect and road performance under the best asphalt content, 5.8%, based on the Marshall design method.

## 4. Conclusions

1. The compaction calculation method of asphalt mixtures during the SGC process was defined based on the multi-phase volume method of granular material mechanics, and the compaction degree was defined by the ratio of the apparent density of the asphalt mixtures to the maximum theoretical density. The relationship equations of compaction degree with the change in the height of the specimen and the maximum theoretical density were also established.
2. The compaction degree prediction equation of the logarithmic function with the amount of compaction was established based on the gyratory compaction curves of SMA. The CEI prediction equation of exponential function with the amount of compaction was established based on CEI curves of SMA. The fitting goodness of these equations was more than 99.8%. These prediction equations can effectively predict the dynamic values of the compaction degree, percentage of air voids, and compaction energy index CEI for the SMA during the SGC process.
3. The compaction process of SMA can be divided into three stages based on the compaction curves rule: Stage I is the rapid growth period, where the compaction degree grows by about 10%, which corresponds to I_1_ times of compaction, and the interval of the number times of compaction is (0, 20); Stage II is the slow growth period, where the compaction degree grows by about 5%, which corresponds to I_2_ times of compaction, and the interval of the number times of compaction is (20, 90); and Stage III is a stable period, where the compaction degree grows by about 1%, and the interval of the number of times of compaction is (90, I_des_), finally trending towards a stable value, with a compaction degree growth of about 15–16% during compaction.
4. The initial compaction temperature has a significant effect on the forming compaction degree of the asphalt mixture; a higher initial compaction temperature increases the mutual lubrication between the asphalt and aggregate, which can effectively improve the compaction degree of the asphalt mixture forming. In gyratory compaction, the optimum compaction temperature of the SMA is about 170 °C, and the effect of the increase in the amount of compaction at a later stage on the increase in the compaction degree of asphalt mixture is very low. During the field compaction, strict attention should be paid to the control of the initial compaction temperature and rolling should be followed closely, which will enable poor compaction quality to be avoided. The initial compaction temperature is recommended as a control indicator of the field compaction process to ensure the compaction quality of the asphalt mixtures.
5. Under the best asphalt content, the friction–lubrication effect between the asphalt and aggregate particles is the best, and can effectively form an asphalt film, reduce the frictional resistance between particles moving with each other in the compaction process, and ensure that the air voids are embedded and filled with each other; thus this degree of compaction is the best. The optimum asphalt content of SMA is about 5.8% with the best compaction degree.

## Figures and Tables

**Table 1:** Physical specifications of coarse aggregate.

Coarse Aggregate Grade (Basalt Gravel)	Apparent Specific Gravity (Dimensionless)	Apparent Density (g/cm^3^)	Bulk Specific Gravity (Dimensionless)	Water Absorption (%)
3–6 mm	2.896	2.889	2.8	1.18
6–11 mm	2.904	2.897	2.818	1.06
11–17 mm	2.906	2.899	2.841	0.78

**Table 2:** Physical specifications of fine aggregate.

Fine Aggregate Grade	Apparent Specific Gravity (Dimensionless)	Apparent Density (g/cm^3^)
0–3 mm	2.680	2.672

**Table 3:** Physical specifications of mineral filler.

Mineral Filler Grade	Apparent Specific Gravity (Dimensionless)	Apparent Density (g/cm^3^)
0–0.6 mm	2.717	2.709

**Table 4:** Physical specifications of fibre.

Name	Specific Gravity (Dimensionless)	Density (g/cm^3^)	Wood Fibre Content (%)
Wood fibre	0.835	0.833	0.4

**Table 5:** Properties of SBS-modified bitumen.

Names of Specifications	Test Results
Specific gravity (dimensionless)	1.024
Density (g/cm^3^)	1.021
Asphalt content (%)	4.8–6.3
Penetration (25 °C, 100 g, 5 s) (0.1 mm)	45.6
Ductility (cm)	35.9
Softening point (R&B) (°C)	76.8

**Table 6:** Aggregate gradation.

Asphalt Mixture Type	Mass Percentage (%) Passing the Following Sieve Sizes (mm)									
	0.075	0.15	0.3	0.6	1.18	2.36	4.75	9.5	13.2	16
SMA-13	9.5	11.9	14.8	17.5	20.2	22.5	25.9	64.3	95.1	100
Upper limit	12	15	16	20	24	26	34	75	100	100
Lower limit	8	9	10	12	14	15	20	50	95	100

## Data Availability

All data generated or analysed during this study are included in this published article.
